# Methods for Enhancing the Formation of Hydroxyl Radicals When Polishing Single Crystal SiC

**DOI:** 10.3390/ma18184276

**Published:** 2025-09-12

**Authors:** Dong Shi, Kaiping Feng, Tianchen Zhao

**Affiliations:** 1 College of Mechanical Engineering, Quzhou University, Quzhou 324000, China; 2 Department of Mechanical & Electrical Engineering, Xiamen University, Xiamen 361005, China

**Keywords:** single crystal SiC, CMP, hydroxyl radical, heterogeneous fenton, tribocatalysis

## Abstract

To enhance the formation of hydroxyl radicals (•OH) when polishing single crystal silicon carbide (SiC), this study proposes a catalytic-assisted polishing approach based on a Fe_3_O_4_/ZnO/graphite hybrid system. Firstly, methyl orange degradation experiments were conducted using Fe_3_O_4_/ZnO/graphite hybrid catalysts. Secondly, a resin-based abrasive tool embedded with the Fe_3_O_4_/ZnO/graphite hybrid was developed. Subsequently, polishing experiments under dry, water, and hydrogen peroxide conditions were performed based on the abrasive tool. The corresponding surface roughness (Sa) were 26.51 nm, 12.955 nm and 4.593 nm, separately. The material removal rate were 0.733 mg/h (1.586 μm/h), 2.800 mg/h (6.057 μm/h) and 4.733 mg/h (10.239 μm/h), respectively. The results demonstrate that the Fe_3_O_4_/ZnO/graphite hybrid synergistically enhanced •OH generation through Fenton reactions and tribocatalysis of ZnO. Therefore, the increased •OH productivity contributes to SiC oxidation and SiO_2_ removal, improving both polishing efficiency and surface finish. The catalytic-assisted polishing provides a novel approach for the high-efficiency ultra-precision machining for SiC.

## 1. Introduction

Single crystal silicon carbide (SiC) exhibits exceptional thermal and electrical properties, making it a promising substrate material for high-temperature, high-frequency, high-power, and radiation-resistant integrated electronic devices [1,2,3]. The rapid expansion of the new energy vehicle market, coupled with the adoption of emerging technologies such as 800 V high-voltage fast charging, has become a critical driver for the accelerated growth of the SiC industry [4]. Increasing wafer size is an effective strategy to reduce costs and enhance production capacity. While 6-inch wafers dominate the current market, 8-inch wafers represent the future direction [5]. Larger substrates enable more chips per wafer, lower per-chip costs, and reduced edge waste, further optimizing cost efficiency. However, the immaturity of global SiC manufacturing and processing technologies has constrained the development of SiC devices [6,7,8]. To fully exploit the superior performance of SiC substrates, advancing high-surface-quality SiC wafer processing technologies is paramount [9].

Chemical mechanical polishing (CMP) is the only method meeting practical requirements of global planarization, atomic-level surface roughness and damage-free surfaces. Although existing techniques for polishing SiC include tribochemical polishing (TCP) [10,11], catalyst-referred etching (CARE) [12], electrochemical mechanical polishing (ECMP) [13,14], and plasma-assisted polishing (PAP) [15], conventional CMP [16] struggles with inefficient chemical reactions when relying solely on oxidants such as hydrogen peroxide (H_2_O_2_) and potassium permanganate (KMnO_4_). Studies by Ni Zifeng et al. [17] showed peak polishing efficiencies of 110 nm/h and 185 nm/h when using H_2_O_2_ and KMnO_4_, respectively. To improve the chemical effect, enhancement strategies utilizing •OH were proposed. Pan Guoshun et al. [18] elucidated the mechanism that •OH can oxidize SiC to form SiO_2_. And UV light [19], laser [20,21], electric fields, and magnetic fields are the main physical methods. For instance, Yan Qiusheng et al. [22,23] demonstrated enhanced catalytic efficiency via electric and magnetic assistance, while Yuan Zewei et al. [24] improved •OH formation using UV photocatalysis.

Currently, the heterogeneous Fenton methods [25] are recommended, which employ solid-phase catalysts to decompose H_2_O_2_ into •OH. Compared to homogeneous Fenton systems, the heterogeneous Fenton methods offers broader pH adaptability, superior catalyst stability, and reusability. In SiC polishing, an Fe_3_O_4_ catalyst exhibits a significant performance enhancement [23,26]. The strategy for improving catalytic efficiency focuses on enhancing Fe^3+^/Fe^2+^ redox cycling and compensating for H_2_O_2_ depletion in real time [27]. The semiconductor of ZnO and electron-rich material of graphite can synergistically promote Fenton reactions by facilitating electron transfer [28]. Additionally, ZnO exhibits tribocatalytic activity, which can degrade RhB dye by generating •OH under mechanical friction [29,30]. Existing research has primarily investigated Fenton catalysis-assisted polishing, in which Fe_3_O_4_ has been proven as a feasible catalyst, as cited in the literature [23]. ZnO and graphene can enhance Fenton catalytic efficiency, as demonstrated in Fenton catalytic decomposition, as cited in the literature [28]. Here, graphene has a mesh-like structure that connects Fe_3_O_4_ and ZnO, providing a pathway for electron transfer, thereby increasing the efficiency of hydroxyl radical formation. In polishing, no exploration of the Fe_3_O_4_/ZnO/graphene composite catalyst has been found, while studies [29,30] have demonstrated the tribocatalytic function of ZnO, facilitating the formation of hydroxyl radicals. Therefore, this paper utilizes the catalytic functionality of Fe_3_O_4_/ZnO/graphene to explore its synergistic enhancement effect in polishing.

To improve •OH formation in CMP, this study integrates heterogeneous Fenton and tribocatalysis to develop a novel Fe_3_O_4_/ZnO/graphite hybrid system for 4H-SiC polishing. Firstly, the catalytic performance of Fe_3_O_4_/ZnO/graphite was investigated. Secondly, the abrasive tool including Fe_3_O_4_/ZnO/graphite was prepared. Finally, the polishing performance of dry polishing, water polishing and hydrogen peroxide polishing based on the abrasive tool were studied separately.

## 2. Experiment and Methods

### 2.1. Abrasive Tool Preparation Process

**Figure 1:**
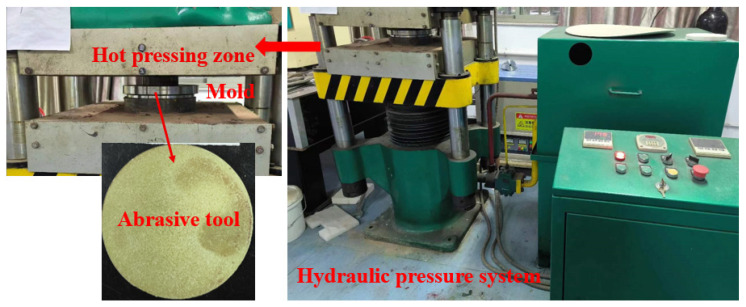
The abrasive tool prepared by the hydraulic pressure system.

The abrasive tools were fabricated using a hot-pressing molding method with a hot-pressing temperature of 180 °C and a holding time of 2 h. The mixed catalyst abrasive tools (dimensions: Φ110 mm × 10 mm) were prepared through the following sequential steps: weighing, mixing, molding, hot-pressing, and demolding. Figure 1 shows the prepared abrasive tool based on the hydraulic pressure system. The abrasive tool structure comprises diamond particles (grit size W2.5), phenolic resin (PR), magnetite (Fe_3_O_4_, 50 nm), zinc oxide (ZnO), graphite, sodium carbonate (Na_2_CO_3_), calcium oxide (CaO), and pores formed by a low-temperature pore-forming agent (PFA). Based on the design principles of abrasive tools, the proportions of abrasive grains, bond, additives, and porosity in the tool were determined. Combined with the usage ratios of Fe_3_O_4_ to ZnO in Fenton catalytic reactions, as referenced in sources [26,28], the final composition ratios of the abrasive tool were determined. The detailed composition ratios are listed in Table 1.

### 2.2. Methyl Orange Degradation Experiment

Based on the principle of hydroxyl radical (•OH)-mediated degradation of methyl orange (refer to the literature), the catalytic efficiency of the catalysts was evaluated by monitoring the decolorization rate of methyl orange. A pH pen (resolution: 0.01) was used to measure and adjust the pH of the methyl orange solution, with the degradation progress assessed via visual color changes. Five experimental groups were designed (see Table 2) using 100 mL of pure water (pH adjusted to 3) under room temperature conditions.

### 2.3. Polishing Experiment

**Figure 2:**
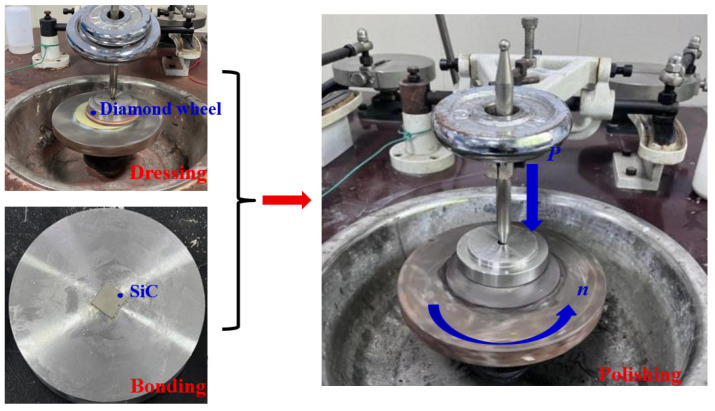
Polishing with the dressed abrasive tool.

As illustrated in Figure 2, the abrasive tool was conditioned using an electroplated diamond grinding wheel. The 4H-SiC samples (dimensions: 12 mm × 12 mm × 500 μm) were bonded to the polishing fixture using paraffin wax. Polishing experiments were conducted on a polishing machine (xibin opto-electronic Co., Ltd, Wuxi, China) with the following parameters: polishing load of 1 kgf (pressure: 68 KPa), rotational speed of 50 rpm, polishing fluid feed rate of 20 mL/h, and polishing time of 1 h. Three types of polishing experiments including dry, water, and hydrogen peroxide polishing were conducted. Each experiment was repeated three times. In the present experimental study, the polished SiC underwent ultrasonic cleaning using analytical-grade ethanol as the solution. The material removal rate (MRR) was calculated using Formula (1) below, and the mass of the silicon carbide was measured using a high-precision electronic balance (resolution: 0.0001 g). Surface morphology and roughness of the polished silicon carbide were analyzed using a super-depth microscope and a laser interferometer.

(1) $$MRR=\frac{\Delta m}{t}\;or\;MRR=\frac{\Delta m}{\rho St}$$

where Δ*m* and *t* represent the mass change and polishing time, ρ (3.21 g/cm^3^) and *S* represent the density and area.

## 3. Results and Discussion

### 3.1. Catalytic Performance of Fe_3_O_4_/ZnO/Graphite Hybrid

**Figure 3:**
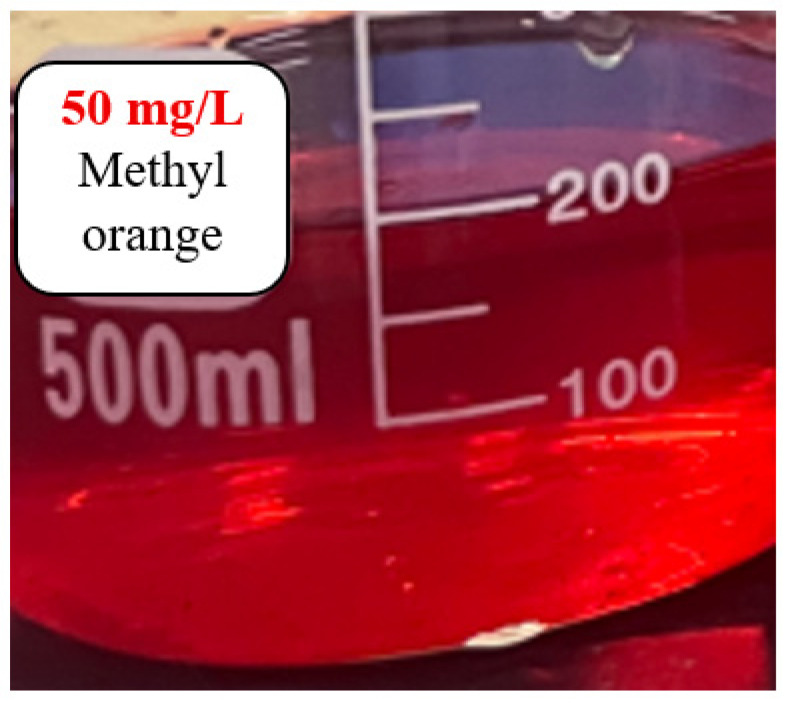
Methyl orange solution (pH3).

**Figure 4:**
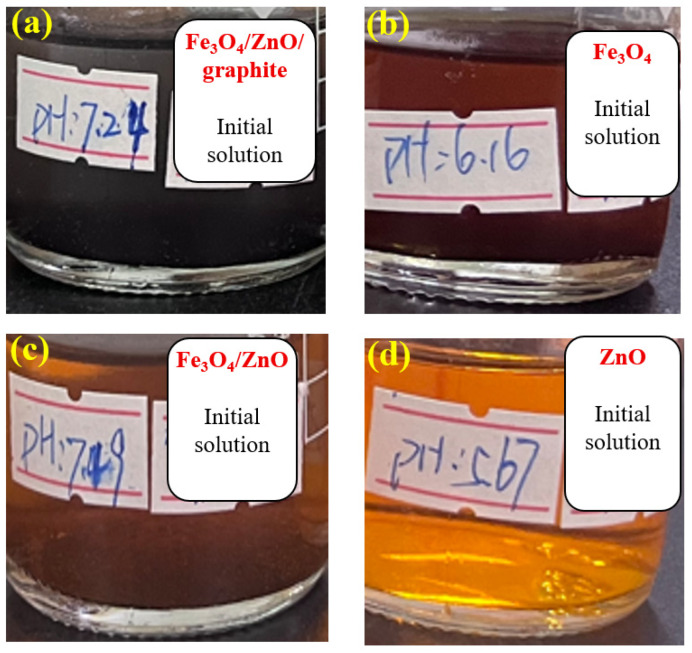
Degradation with different catalysts: (**a**) Fe_3_O_4_/ZnO/graphite; (**b**) Fe_3_O_4_; (**c**) Fe_3_O_4_/ZnO; (**d**) ZnO.

**Figure 5:**
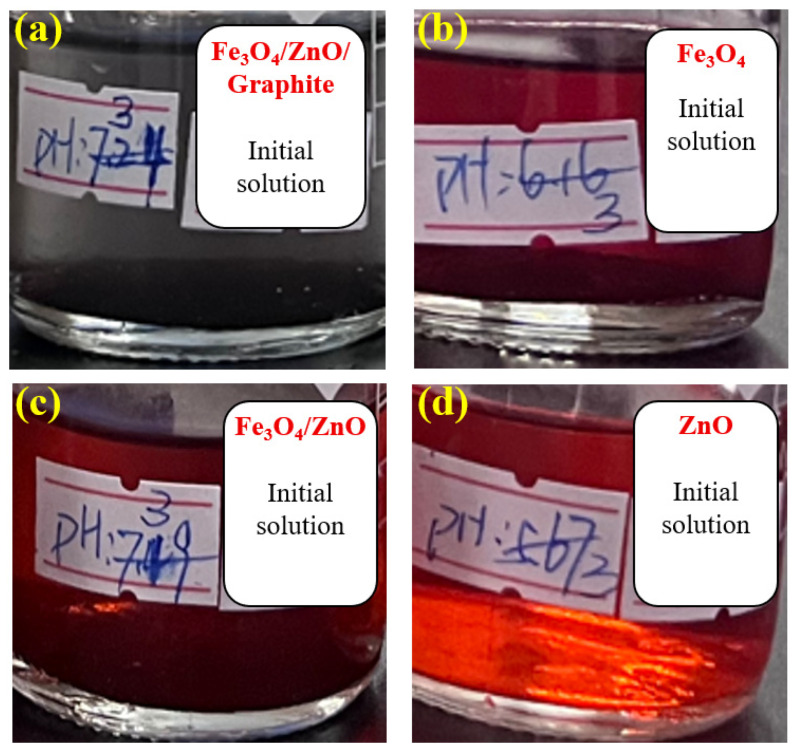
Solution colors at pH3: (**a**) Fe_3_O_4_/ZnO/graphite; (**b**) Fe_3_O_4_; (**c**) Fe_3_O_4_/ZnO; (**d**) ZnO.

Figure 3 shows the methyl orange solution of the control group (without any catalyst), which appeared red after being placed at pH 3 for 1 h, consistent with the color-changing principle of methyl orange. As a commonly used acid-base indicator, methyl orange exhibits a red color at pH values below 3.1. Within the range of 3.1 to 4.4, the red color gradually fades, turning to orange-yellow or pale yellow near pH 4.4; above pH 4.4, it appears yellow. Figure 4 displays the pH values and colors of the solutions after 12 h with four types of catalysts which are Fe_3_O_4_/ZnO/graphite, Fe_3_O_4_, Fe_3_O_4_/ZnO, and ZnO, respectively. The corresponding pH values were 7.24, 6.16, 7.49 and 5.67, and the colors are colorless, light red, orange-yellow and yellow, respectively. The increase in pH during the degradation process is attributed to the generation of hydroxide ions during catalysis. According to the color-changing principle of methyl orange, the colorless and transparent solution in Figure 4a suggests that methyl orange may have been fully degraded. Figure 5 shows the solutions from Figure 4 after adjusting the pH back to 3. The mixed catalyst solution remained colorless and transparent, while the others turned red. This further confirms that methyl orange was fully degraded in the mixed catalyst solution. By combining the observations from Figure 4 and Figure 5, it can be concluded that the mixed catalyst achieved complete degradation of methyl orange, demonstrating the highest catalytic efficiency among the four types of catalysts.

### 3.2. Polishing Performance Using Catalytic Abrasive Tool

**Figure 6:**
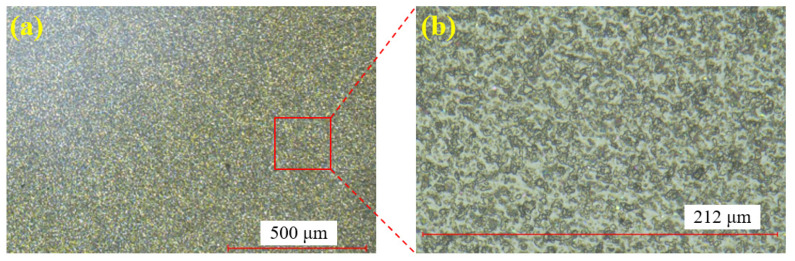
SiC surface before polishing: (**a**) at 100× magnification; (**b**) zoomed-in view.

Figure 6 shows the single crystal SiC sample before polishing. Figure 6a displays the surface at 100× magnification, while Figure 6b provides a zoomed-in view of Figure 6a. Through the super-depth microscope (SDM), it is evident that the surface after wire cutting is rough, with numerous brittle pits present.

**Figure 7:**
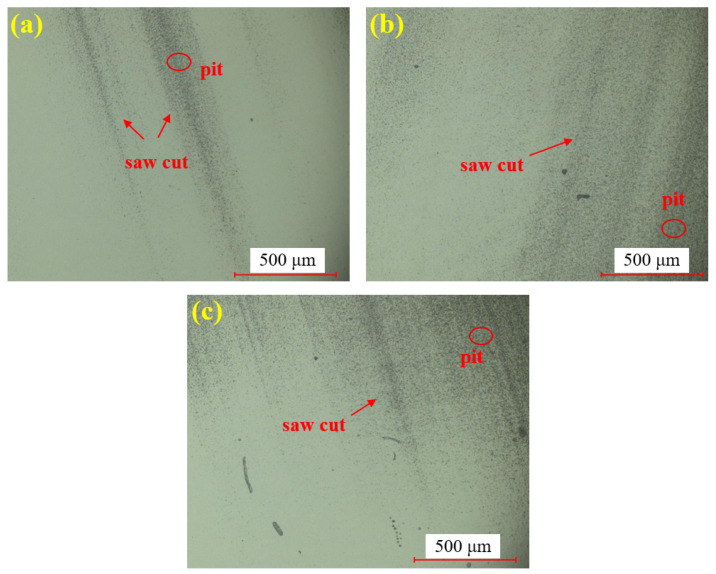
SDM images by dry polishing: (**a**) sample 1; (**b**) sample 2; (**c**) sample 3.

Figure 7 presents the dry polishing results of single crystal SiC under the super-depth microscope. Figure 7a–c corresponds to three repeated experiments using the mixed abrasive tool under the same polishing conditions. Compared to the original sample, the surfaces of the three SiC samples after dry polishing show a significant reduction in brittle pits, with a notable improvement in surface roughness. However, some pits remain unremoved on all three samples, and the residual scratches exhibit a distinct directionality. This is attributed to the deep saw marks left on the SiC surface after wire cutting, combined with the relatively low material removal rate of dry polishing, which prevents the complete removal of brittle scratches in the deeper saw marks.

**Figure 8:**
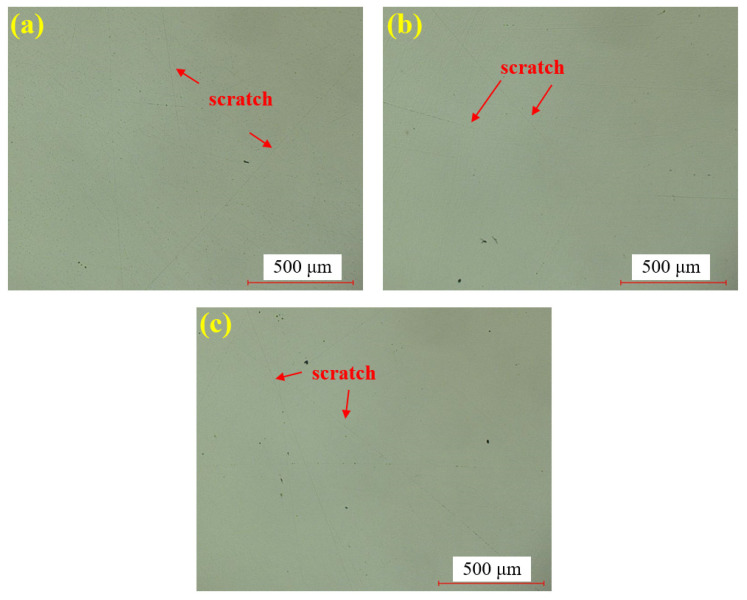
SDM images by water polishing: (**a**) sample 1; (**b**) sample 2; (**c**) sample 3.

Figure 8 shows the water polishing results of single crystal SiC under the super-depth microscope. Figure 8a–c represents three repeated experiments using the mixed abrasive tool under the same polishing conditions. Compared to the original sample, no brittle scratches are observed on the silicon carbide surface, and the surface roughness is significantly improved. In comparison to the dry-polished samples, the residual saw marks on the silicon carbide surface have been removed. Based on this, it can be preliminarily concluded that the efficiency of water polishing is higher than that of dry polishing.

**Figure 9:**
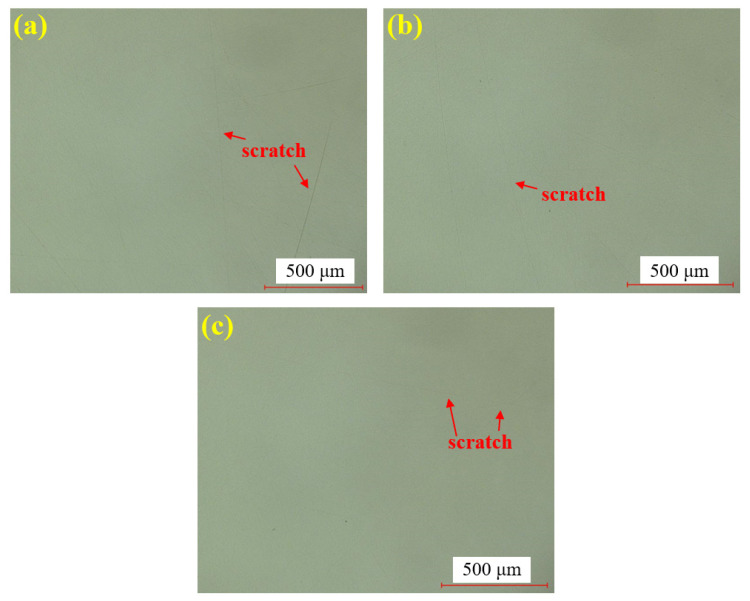
SDM images by hydrogen peroxide polishing: (**a**) sample 1; (**b**) sample 2; (**c**) sample 3.

Figure 9 presents the hydrogen peroxide polishing results of single crystal SiC under the super-depth microscope. Figure 9a–c corresponds to three repeated experiments using the mixed abrasive tool under the same polishing conditions. Similarly to the water-polished samples, no brittle scratches are observed on the silicon carbide surface, and the residual saw marks have been removed, with a significant improvement in surface roughness. Similarly, it can be concluded that the polishing efficiency is higher than that of dry polishing.

**Figure 10:**
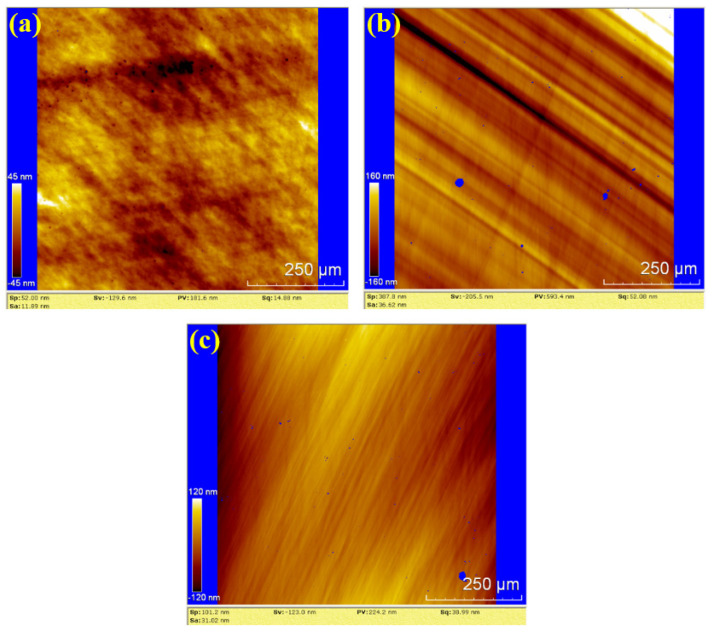
WLI images by dry polishing: (**a**) sample 1; (**b**) sample 2; (**c**) sample 3.

**Figure 11:**
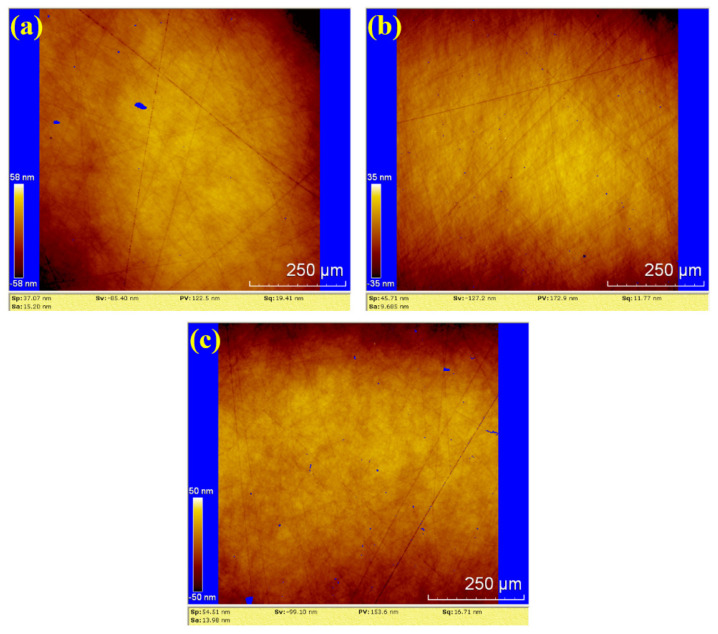
WLI images by water polishing: (**a**) sample 1; (**b**) sample 2; (**c**) sample 3.

**Figure 12:**
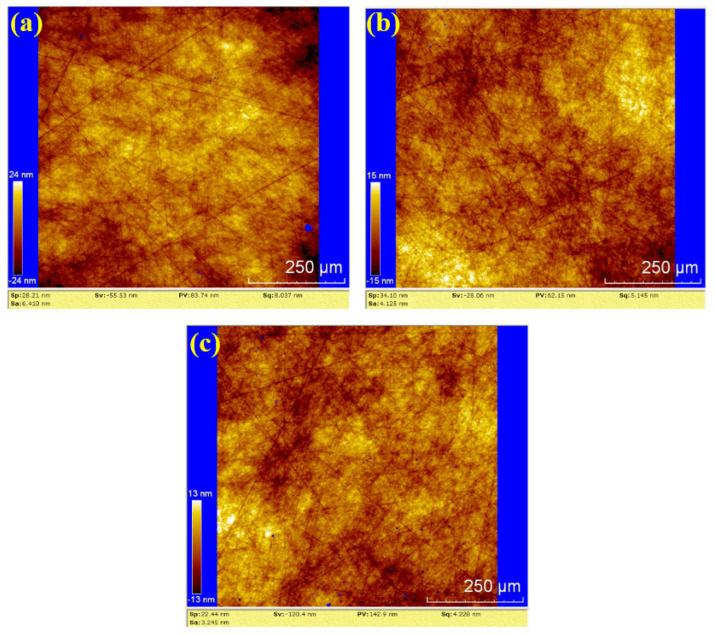
WLI images by hydrogen peroxide polishing: (**a**) sample 1; (**b**) sample 2; (**c**) sample 3.

To further evaluate the surface roughness and topography, white light interferometry (WLI) was used to analyze the polished surfaces. The results for dry polishing, water polishing, and hydrogen peroxide polishing are shown in Figure 10, Figure 11 and Figure 12, respectively. In Figure 10, the surface roughness values (Sa) for the three samples are 11.89 nm, 36.62 nm and 31.02 nm, The corresponding peak-to-valley (PV) values are 181.6 nm, 593.4 nm, and 224.2 nm, respectively. The average values for dry polishing are Sa 26.51 nm and PV 333.07 nm. Figure 10a reveals residual scratches and pits on the sample surface, while Figure 10b,c shows distinct directional scratches, similar to the grinding marks left by a grinding wheel.

In Figure 11, the surface roughness values (Sa) for the three samples are 15.20 nm, 9.685 nm and 13.98 nm. The corresponding PV values are 122.5 nm, 172.9 nm and 153.6 nm, respectively. The average values for water polishing are Sa 12.955 nm and PV 149.67 nm. Compared to the dry polishing results, the surfaces of the three water-polished samples exhibit random scratches with shallow depths, and no residual saw marks. Therefore, the surface roughness of water polishing is lower than that of dry polishing, and the surface quality is superior to dry polishing.

In Figure 12, the surface roughness values (Sa) for the three samples are 6.410 nm, 4.125 nm, and 3.245 nm. The corresponding PV values are 83.74 nm, 62.15 nm, and 142.9 nm, respectively. The average values for hydrogen peroxide polishing are Sa 4.593 nm and PV 96.263 nm. Compared to the water polishing results, the depth of random scratches on the three samples is reduced, but the number of scratches increased. This suggests that the number of effective abrasive particles in the hydrogen peroxide polishing process is higher than in water polishing, resulting in lower pressure exerted by each abrasive particle on the silicon carbide under the same load. Consequently, the average roughness and PV values are lower than those of water polishing.

**Figure 13:**
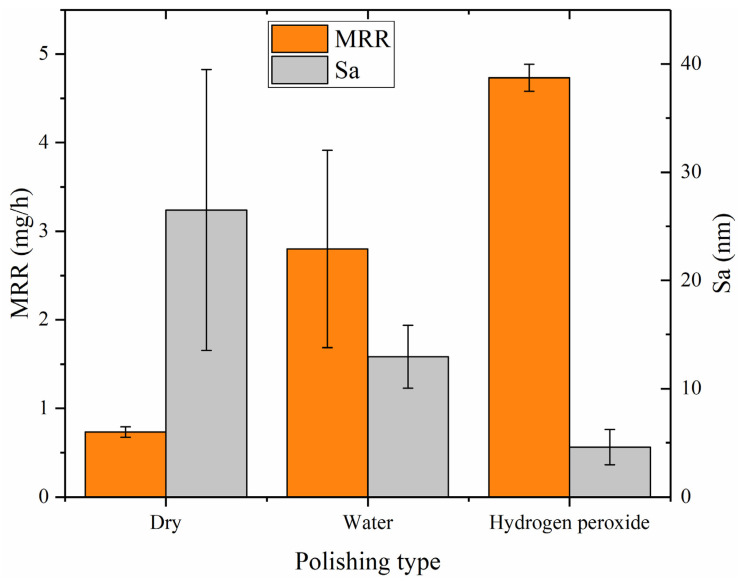
The MRR (left vertical axis) and surface roughness (right vertical axis).

Based on statistical analysis, Figure 13 (a dual *Y*-axis plot) displays averages and deviations of the surface roughness (Sa) and MRR for dry polishing, water polishing, and hydrogen peroxide polishing. The left vertical axis represents the MRR (unite: mg/h), while the right is the surface roughness (unite: nm). The graph illustrates that hydrogen peroxide polishing exhibits the best performance, with a surface roughness of Sa 4.593 nm and a material removal rate of 4.733 mg/h (10.239 μm/h). Dry polishing shows the lowest performance, with a surface roughness of Sa 26.51 nm and a material removal rate of 0.733 mg/h (1.586 μm/h). Water polishing falls in between, with a surface roughness of Sa 12.955 nm and a material removal rate of 2.800 mg/h (6.057 μm/h). The low material removal rate of dry polishing results in residual saw marks on the surface, leading to a larger standard deviation in surface roughness among the three polishing methods. Both water polishing and hydrogen peroxide polishing achieve higher material removal rates, completely eliminating the saw marks on the silicon carbide surface and resulting in lower surface roughness. As the depth of surface scratches decreases, the mechanical action of the abrasive particles during water polishing and hydrogen peroxide polishing is reduced. However, the increased efficiency suggests that the catalysts in the abrasive tools are playing a role, with the catalytic chemical effects compensating for the reduced mechanical effects. Hydrogen peroxide polishing demonstrates the highest efficiency, indicating the highest catalytic efficiency and the most effective oxidation of silicon carbide by hydroxyl radicals.

### 3.3. Material Removal Mechanism of SiC Polishing Using Catalytic Abrasive Tool

**Figure 14:**
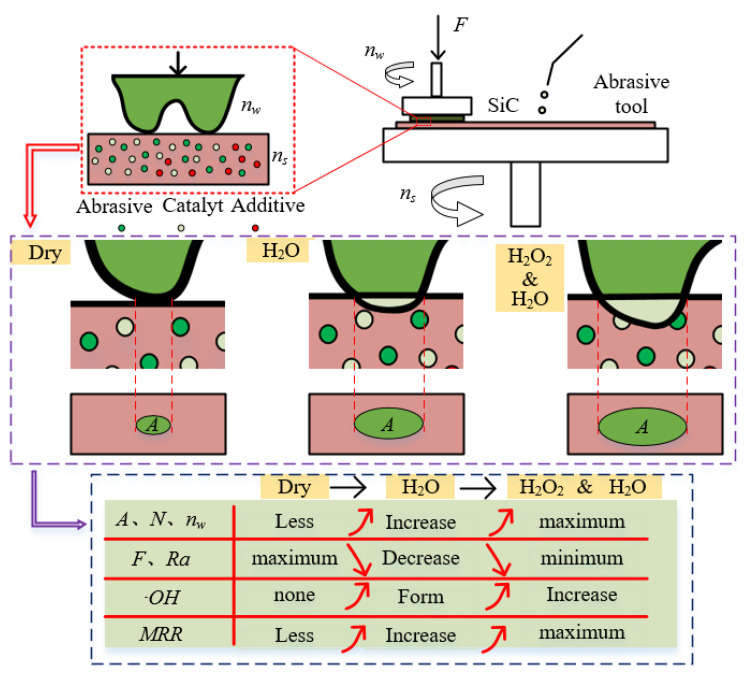
Polishing mechanism using catalytic abrasive tool under different conditions.

Based on the results and analysis of methyl orange degradation and single crystal silicon carbide polishing, Figure 14 illustrates the mechanism of the mixed catalyst abrasive tool for dry polishing, water polishing, and hydrogen peroxide polishing of single crystal silicon carbide. In dry polishing, the interaction between the silicon carbide micro-protrusions and the abrasive tool is primarily mechanical. In contrast, water polishing and hydrogen peroxide polishing involve chemical reactions, leading to differences in the contact area (A), the number of effective abrasive particles (N), and the force per abrasive particle (F) between the silicon carbide and the abrasive tool. Dry polishing results in a smaller contact area, fewer effective abrasive particles, and a larger force per abrasive particle, which increases the scratch size and reduces the silicon carbide rotational speed (nw), resulting in poor surface quality and a low MRR. In water polishing and hydrogen peroxide polishing, catalytic oxidation reactions occur, increasing the removal efficiency of the silicon carbide micro-protrusions. This leads to an increase in the contact area, the number of effective abrasive particles, and the silicon carbide rotational speed, while reducing the force per abrasive particle. As a result, the material removal rate is higher, and the surface quality is improved. Since hydrogen peroxide enhances catalytic efficiency, the formation rate of ·OH during polishing increases, further improving the oxidation efficiency of SiC. Therefore, hydrogen peroxide polishing exhibits the highest efficiency and the best surface quality.

## 4. Conclusions

This study proposes a multiphase catalytic-assisted polishing method for single crystal SiC by combining heterogeneous Fenton and tribocatalysis. Through systematic experiments on methyl orange degradation and SiC polishing using a hybrid catalyst-integrated abrasive tool, the following conclusions are drawn:

(1) The Fe_3_O_4_/ZnO/graphite hybrid catalyst demonstrated exceptional •OH generation efficiency, enabling complete methyl orange degradation under optimized conditions. Integration of this catalyst into SiC polishing systems enhances •OH oxidation, significantly boosting the MRR.
(2) A phenolic resin-based abrasive tool embedded with Fe_3_O_4_/ZnO/graphite was successfully fabricated via hot-pressing at 180 °C for 2 h, providing a scalable platform for catalytic-assisted polishing.
(3) Water polishing achieved an MRR of 2.800 mg/h (6.057 μm/h) and a surface roughness of Sa 12.955 nm. Hydrogen peroxide polishing achieved an MRR of 4.733 mg/h (10.239 μm/h) and Sa 4.593 nm, which is the highest efficiency and best surface quality. Mechanistic analysis confirmed that catalytic •OH generation reduced mechanical scratch depth while increasing oxidation-driven material removal.
(4) Compared to dry polishing, water and hydrogen peroxide polishing reduced surface roughness by 51% and 83%, respectively, while increasing MRR by 282% and 546%, demonstrating their potential for high efficiency polishing of SiC.
(5) Based on the Fe_3_O_4_/ZnO/graphite abrasive tool, further research into the atomic-level polishing processes of water polishing and hydrogen peroxide polishing holds promise for developing an efficient, ultra-precise, and environmentally friendly polishing method.

## Figures and Tables

**Table 1:** Composition ratio of abrasive tool.

Component	Proportion (%)	Mass (g)
Diamond	20	12
PR	30	18
Fe_3_O_4_	5	3
ZnO	5	3
Graphite	5	3
Na_2_CO_3_	20	12
CaO	8	4.8
PFA	7	4.2

**Table 2:** Experimental design.

Reagent	Control Group	a	b	c	d
Methyl Orange (mg)	5	5	5	5	5
30% H_2_O_2_ (mL)	0	5	5	5	5
Fe_3_O_4_ (g)	0	1	1	1	0
ZnO (g)	0	1	0	1	1
Graphite (g)	0	1	0	0	0

## Data Availability

The original contributions presented in this study are included in the article. Further inquiries can be directed to the corresponding author.
